# Nano-Hydroxyapatite and Calcium-Enriched Mixture for Pulp Capping of Sound Primary Teeth: A Randomized Clinical Trial

**Published:** 2015-03-18

**Authors:** Roza Haghgoo, Saeed Asgary, Fatemeh Mashhadi Abbas, Roshanak Montazeri Hedeshi

**Affiliations:** a *Department of Pediatric Dentistry, Dental School, Shahed University, Tehran, Iran; *; b * Iranian Center for Endodontic Research, Research Institute of Dental Sciences,Dental School, Shahid Beheshti University of Medical Sciences, Tehran, Iran; *; c * Department of oral Pathology, Dental school, Shahid Beheshti University of Medical Sciences, Tehran, Iran; *; d * Department of Pediatric Dentistry, Dental School, Qom University of Medical Science, Qom, Iran*

**Keywords:** Calcium-Enriched Mixture, Direct Pulp Capping, Nano-Hydroxyapatite, Primary Teeth, Vital Pulp Therapy

## Abstract

I**ntroduction: **Nano-hydroxyapatite (NHA) has been used for regeneration of osseous defects. Calcium-enriched mixture (CEM) cement is also used for various dental treatments. This trial compared the efficacy of NHA and CEM cement for direct pulp capping (DPC) of sound primary teeth. **Methods and Materials: **In this randomized clinical trial with split-mouth design, after attaining informed consent, 20 sound primary canines scheduled for orthodontic extraction, were selected. After mechanical pulp exposure, the exposed site was capped with either NHA or CEM cement and then immediately restored with glass-ionomer and resin composite. The teeth were extracted after two months and examined histologically. Parameters of hard tissue bridge (HTB) formation, its type and quality as well as pulpal inflammation scores were compared between the two experimental groups. The data were analyzed using the Mann Whitney U and Fisher’s exact test. The level of significance was set at 0.001. **Results: **All CEM specimens showed inflammation score of 0 (less than 10%). However, in NHA group, inflammation scores of 0 (less than 10%), 1 (10%-30%) and 2 (30%-50%) were observed in 2 (20%), 4 (40%) and 4 (40%) specimens, respectively (*P*<0.001). HTB was formed in all CEM specimens while it was developed in 2 specimens of NHA (20%; *P*<0.001). All CEM specimens showed normal pulp; only two cases in NHA group (20%) demonstrated uninflamed normal pulp. **Conclusion: **CEM cement was superior to NHA as a DPC agent in terms of HTB formation and pulp inflammation scores. It is a suitable material for the DPC of primary teeth.

## Introduction

Direct pulp capping (DPC) of primary teeth is not suggested in the literature due to the high cellularity of pulp that ultimately leads to treatment failure [1, 2]. Undifferentiated mesenchymal cells may differentiate into odontoclasts and cause internal resorption. Thus, DPC has a poor prognosis in primary teeth due to the high risk of internal resorption, acute alveolar abscess, risk of pulpal calcification, necrosis and trauma to the adjacent bone [3, 4]. However, DPC is appealing to clinicians and researchers because of its conservative nature [5] and positive results for DPC with formation of normal dentin in treatment of carious pulp exposures has been reported [1, 2]. 

Several materials are used as DPC agents. For many years, calcium hydroxide (CH) used to be the standard of choice for the DPC of vital teeth [6]. CH has antimicrobial properties and can induce the formation of a microscopic calcified bridge. This bridge however, has some drawbacks and does not provide permanent seal so it may allow bacterial leakage [7]. Based on the literature, the success rate of DPC in primary teeth with CH is not considerably high [8]. Based on the long-term evidence, CH is dissolved over time and allows bacterial microleakage; consequently, pulp inflammation and necrosis occurs within 1-2 years [9]. Thus, using some other biomaterials for this purpose seems necessary. Hydroxyapatite (HA), tricalcium phosphate, mineral trioxide aggregate (MTA), bone morphogenic protein (BMP), *etc.* have been used for this purpose [3, 10-13]. 

Despite of being highly biocompatible, MTA has some drawbacks such as long setting time, difficult handling and high cost [14]. Calcium-enriched mixture (CEM) cement contains calcium compounds with antimicrobial properties. It is biocompatible [15], has the ability to induce the formation of HA crystals and HTB [16], can be effectively used for treatment of furcal perforation in the primary teeth [17] and for vital pulp therapy (VPT) in primary [18] as well as permanent teeth [19-21]. Several clinical trials have evaluated the effects of CEM cement on human dental pulp; its favorable properties are strengthened in wet environment and the biomaterial seems to be suitable for VPT [22, 23].

Calcium phosphate cements (CPC) have been used in dentistry and orthopedics for more than 40 years due to their similarity to the mineral phase of natural bone [24]. HA is a remineralizing agent for application to the enamel surface [25]. Due to optimal characteristics such as antibacterial properties, high similarity to the mineral tissue, biocompatibility and low solubility, it is widely used for biological, medical and dental purposes [26]. HA has numerous applications in restoration, regeneration and reconstruction of bony defects [24]. Several studies have tried to improve the characteristics, bioactivity, mechanical strength and solubility of HA by controlling its composition, morphology and size of nanoparticles [27]. Nanotechnology plays an important role in development of porous bioceramics with high mechanical strength, optimal bioactivity and increased solubility [28]. Nanotechnology has demonstrated that nano-scale materials often show different and sometimes more favorable physical, chemical and biological properties than their mass form [29]. 

Calcium and phosphate molecules are manipulated at the molecular level and combined in order to produce biomaterials with specific structure and functional properties [30]. Nano-phase calcium phosphate can mimic the dimensions of the natural tissue components and increase osteoblastic adhesion and resorption in genetically engineered tissue implants [31]. Nano-Hydroxyapatite (NHA) is a bioactive and biocompatible material with extensive applications in medicine and dentistry. It has gained acceptance due to having greater contact area and higher solubility compared to HA [32]. Some studies have investigated the remineralizing effect of NHA on demineralized enamel and dentin lesions [26]. 

Direct effects of NHA have also been investigated on humans in some clinical trials [33]. However, its effect on human dental pulp has never been evaluated before. Moreover, only limited studies have qualitatively evaluated the efficacy of different DPC agents on pulps of primary teeth [34]. This randomized clinical trial aimed to compare the histological effects of NHA and CEM cement on pulps of primary teeth after DPC.

## Materials and Methods

This Randomized clinical trial with split mouth design was conducted on 20 sound primary canines belonging to 10 children (2 in each patient) that had to be extracted according to their orthodontic treatment plan. The sample size was calculated to be 19 subjects via the conduction of a pilot study taking into account the power of 85% and confidence interval of 95%. 

Parents of children were thoroughly aware of the study design and signed an informed consent. The teeth were divided into 2 groups (*n*=10). All teeth had sound crowns and their root resorption (if present) was confined to the apical third of the root. The exclusion criteria were systemic complications, taking medications, spontaneous or nocturnal toothache and uncooperative children. The selected teeth did not have internal or pathological external root resorption, apical or inter-radicular radiolucency, physiological or pathological mobility, abscess, sinus tract, periodontal pocket, spontaneous pain or regional lymphadenopathy. 

After local anesthesia, class V cavities were prepared 1 mm above the gingival margin using a carbide bur under irrigation with water. Cavity preparation was continued until a shadow of dental pulp appeared. After irrigation with saline and drying the cavity, a 0.5-0.7-mm mesiodistal exposure was created at the center of the cavity using the sharp tip of a sterile explorer. The cavity was rinsed with saline solution and dried with cotton pellet. After achieving hemostasis at the exposure point, the DPC agent was applied. In each child, CEM cement (BioniqueDent, Tehran, Iran) was used for one primary canine and NHA (Iran Polymer and Petrochemical Institute) for another. CEM cement and NHA were prepared according to the manufacturers’ instructions. 

Randomization process was as follows: letters A and B were written on pieces of papers and placed in a jar. After preparing the first primary canine, a piece of paper was taken out and based on the letter, the patient was assigned to the respective group. The teeth were eventually restored with a layer of light-cure glass ionomer (Fuji II LC, GC Corporation, Tokyo, Japan) and composite resin (Heliomolar HB; Ivoclar Vivadent, Schaan, Liechtenstein, Austria) [35].

The teeth were extracted 60 days later, fixed in 10% formalin for 48 h and decalcified in 10% nitric acid and 14% diamino tetraacetic acid. The teeth were then embedded in paraffin and cut with a microtome in 5-µm slices. The sections were stained with Hematoxylin and Eosin (H&E). Samples were evaluated microscopically by a pathologist who was blind to the study using a light microscope (Olympus SZ, Germany) and 40×, 100× and 200× magnification. Presence or absence of inflammation, degree of inflammation, presence of odontoblastic layer and the appearance of HTB (not formed, complete HTB, partial HTB) were reported for each specimen. The degree of inflammation was scored as follows: *score 0*- less than 10%; *score 1*- 10%-30%; *score 2*- 30%-50% and *score 3*-more than 50% [36].

HTB formation and degree of inflammation were compared between the two groups using the Mann Whitney U and Fisher’s exact tests. The level of significance was set at 0.001.

## Results

HTB was formed in all CEM specimens (100%); which was irregular in all cases. HTB was not formed in 8 (80%) out of 10 teeth in NHA (Table 1). HTB in the 2 remaining specimens was irregular. The Fisher’s exact test revealed significant differences between the two groups in this regard; the frequency of HTB formation was significantly higher in CEM group compared to NHA specimens (*P*<0.001).

In terms of pulp inflammation, all CEM specimens (100%) showed inflammation score of 0, but in NHA group, inflammation scores of 0, 1 and 2 were observed in 2 (20%), 4 (40%) and 4 (40%) specimens, respectively. According to the chi-square test, the inflammation was significantly higher in the NHA group (*P*<0.001) (Table 2).

## Discussion

This randomized controlled clinical trial compared the formation of HTB and pulp inflammation subsequent to DPC of sound primary molars with CEM cement and NHA. The results revealed that in both fields CEM cement showed much better results than the NHA.

Attempts have been made to introduce new materials for DPC. Evidence shows that the reaction of pulp to restorative materials is transient and severe inflammation only occurs when the microorganisms and bacterial products enter the pulp [9]. The frequency of healthy odontoblastic layer in CEM specimens is significantly higher than NHA specimens; which can be attributed to the type of pulp capping material that can damage the odontoblastic layer [37]. 

**Table 1:** The formation [N (%)] of hard tissue bridge (HTB)

**Group**	**HTB formation**	
	**Yes**	**No**
**CEM**	10 (100)	0
**NHA**	2 (20)	8 (80)

In a study by Shayegan *et al*. [4], moderate or severe inflammatory reactions were not seen following pulpotomy and DPC of swine teeth with NHA and only one specimen in the pulpotomy and DPC groups showed signs of a compact layer with moderate inflammation below the exposed site. In their study, NHA induced soft tissue formation in the pulp of primary swine teeth. In this regard, the results of the present human study do not match. Controversy may be attributed to the application of different types of NHA crystals (type of crystals and size of particles) and different preparation methods.

In a study by Subay and Asci [5], HA could not induce HTB formation at the exposed areas of human dental pulp and no sign of HTB formation was seen in the exposed areas of teeth that underwent DPC with HA. This finding is in agreement with our results. In another study by Su *et al. *[38], the pulpal reaction to biological NHA composites applied as DPC agent was evaluated in dog teeth and it was revealed that NHA had optimal biocompatibility but could not adequately induce the formation of a restorative dentinal HTB compared to CH. Our results confirmed these findings. 

The fabrication methods of NHA include plasma, incremental hydrolysis of calcium phosphate salts, sol-gel technique and crystal growth under hydrothermal conditions [39]. For instance, evidence shows that apoptosis or programmed cell death, destruction of cell morphology, release of cell necrosis factors and subsequent induction of inflammatory responses may significantly increase in response to the application of two different types of NHA crystals by decreasing the length and diameter of crystals by approximately 7 nm and 20% reduction in degree of crystallization. Particles present in NHA are highly motile [40]. Thus, NHA may act as chemical absorbent for cells with filopodia due to selective absorption of some proteins. Since collagen enhances the ability of osteoblasts for adhesion, NHA facilitates the invasion of osteoblasts. During the process of bone regeneration after injury, the inflammation phase occurs following the healing phase and manifests by the formation of hard scar during matrix mineralization. Within 3-6 weeks, the newly formed bone becomes trabecular and this event can be histologically observed [4]. In the study by Shayegan *et al.* [4] NHA showed great potential for healing after the inflammation phase in pulpotomized teeth.

Application of CEM cement and MTA for DPC cause faster formation of a more uniform HTB; however, the HTB induced by CH is usually not complete [41]. There is a rare case report of HTB formation below the CEM cement and successful apexogenesis after 1 month subsequent to pulpotomy of an immature traumatized maxillary central incisor [42]. In the present study all specimen in CEM cement group had formed HTB which was irregular in appearance.

**Table 2:** The degree of inflammation [N (%)]

**Group**	**Degree of inflammation**			
	**0 (<10%)**	**1 (10%-30%)**	**2 (30%-50%)**	**3 (>50%)**
**CEM**	10 (100)	0 (0)	0 (0)	0 (0)
**NHA**	2 (20)	4 (40)	4 (40)	0 (0)

CEM cement contains calcium oxide. During the process of hydration, CH is formed; which has the ability to induce formation of HTB [41]. It has suitable properties that has the ability to induce the formation of HTB and shorter setting time; which are optimal for DPC treatment [16]. 

Formation of the HTB at the pulp-material interface is a controversial subject; because the presence of HTB does not necessarily indicate a healthy pulp tissue. It does not protect the pulp from bacterial microleakage either. However, it can still be a sign of recovery or reaction to irritation [41, 43]. HTB was thicker in CEM specimens and had a more tubular pattern along with a palisading pattern of odontoblast or odontoblast-like cells indicating that HTB in CEM specimens gradually becomes calcified and reaches an adequate thickness (less than 0.25 mm) [41, 44]. CEM cement is superior to CH in many ways [45]. It induces the formation of a complete HTB thicker than that of CH. Presence of odontoblast-like cells beneath the HTB after DPC with CEM has been confirmed [45]. Favorable biocompatibility of CEM may be due to its chemical composition. It has the ability to produce HA [1] which is a natural component for dental pulp cells. The composition of all materials released from CEM is not clear but it contains CH and calcium oxide; and release of adequate amounts of CH is sufficient to induce a calcific response from the directly capped tissue [46]. 

It seems that the calcium sulfate and calcium silicate component of CEM cause its limited expansion following continuous hydration after its initial setting and lead to its further crystalline maturation.

## Conclusion

CEM cement is superior to nano-hydroxyapatite for direct pulp capping of primary teeth. However, further investigations on teeth with previous carious lesions are required. Considering the newly introduced biocompatible compounds, pulp treatment of primary teeth may undergo a paradigm shift in future.
